# Establishment, characterization and functional testing of two novel ex vivo extraskeletal myxoid chondrosarcoma (EMC) cell models

**DOI:** 10.1007/s13577-022-00818-x

**Published:** 2022-11-01

**Authors:** Jana Lucia Bangerter, Kim Jannis Harnisch, Yanjiang Chen, Catherine Hagedorn, Lara Planas-Paz, Chantal Pauli

**Affiliations:** 1 Department of Pathology and Molecular Pathology, University Hospital Zurich, Schmelzbergstrasse 12, 8006 Zurich, Switzerland; 2 Medical Faculty, University of Zurich, Zurich, Switzerland

**Keywords:** Extraskeletal myxoid chondrosarcoma, Ex vivo cell model, Sarco-sphere, Functional testing, Molecular profiling

## Abstract

Extraskeletal myxoid chondrosarcoma (EMC) is a malignant mesenchymal neoplasm of uncertain differentiation as classified by the WHO Classification of Tumours 2020. Although often associated with pronlonged survival, EMC has high rates of distant recurrences and disease-associated death. EMCs are translocation sarcomas and harbor in > 90% of the cases an *NR4A3* rearrangement. The molecular consequences of the *NR4A3* gene fusions are not yet fully elucidated as well-characterized ex vivo cell models for EMC are lacking. Patient-derived ex vivo models are important and essential tools for investigating disease mechanisms associated with diseases that are rare, that exhibit poor prognosis and for the identification of potential novel treatment options. We established two novel EMC ex vivo models (*USZ20-EMC1 and USZ22-EMC2*) for functional testing and research purposes. *USZ20-EMC1* and *USZ22-EMC2* were established and maintained as sarco-sphere cell models for several months in culture. The cells were molecularly characterized using DNA sequencing and methylation profiling. Both cell models represent their native tumor tissue as confirmed by histomorphology and their molecular profiles, suggesting that native tumor cell function can be recapitulated in the ex vivo models. Using a functional screening approach, novel anti-cancer drug sensitivities including potential synergistic combinations were identified. In conclusion, two novel EMC ex vivo cell models (*USZ20-EMC1 and USZ22-EMC2*) were successfully established and characterized from native tumor tissues. Both cell models will be useful tools for further investigating disease mechanisms and for answering basic and translational research questions.

**Supplementary Information:**

The online version contains supplementary material available at 10.1007/s13577-022-00818-x.

## Introduction

Soft tissue sarcomas (STS) are rare tumors of mesenchymal origin, accounting for < 1% of all cancers. They are very diverse and are comprised of greater than 75 distinct histopathological subtypes [1]. In general, these tumors are often difficult to diagnose and therapeutic options are limited. Preclinical cancer model systems have become indispensable for molecular target discovery and to prioritize drugs and drug combinations [2]. However, due to the rarity, histologic and genomic heterogeneity as well as sarcoma histotype missclassifications, there is often a lack of ex vivo sarcoma models. Extraskeletal myxoid chondrosarcoma (EMC) is an exceptionally rare subtype with uncertain differentiation, accounting for < 1% of STS. Despite its name, there is no evidence of cartilaginous differentiation [3]. EMC arise most often in the deep soft tissue of proximal extremities and limb girdles, with the thigh being the most common site. EMC usually occur in adults, with a median age of 50 years [4]. EMC form large, well-demarcated tumors with a multinodular architecture and contain fibrous septa that divide the tumor into hypocellular lobules with abundant pale-blue myxoid or chondromyxoid matrix. From a genetic standpoint, EMC typically harbor chromosomal translocations involving the Nuclear Receptor Subfamily 4 Group A (*NR4A3*) gene, in a near-diploid karyotype. The vast majority of cases (> 70%) show *NR4A3* fused to *EWSR1* (Ewing Sarcoma RNA Binding Protein 1) and less frequently (approximately 20%) to *TAF15* (TATA-Box Binding Protein Associated Factor 15) [5]. Other rare new fusion partners for *NR4A3* have also been reported (e.g., *HSPA8*) [6]. Although often associated with prolonged survival, EMC have high rates of distant recurrences and disease-associated death. Metastases are usually pulmonary but can also disseminate to extrapulmonary locations. Metastatic patients with non-resectable disease and with evidence of tumor progression need a systemic therapy. Anthracycline-based chemotherapy, which is the first-line regimen used in STS, has low activity in this sarcoma subtype [7, 8]. Some evidence exists that antiangiogenic drugs such as sunitinib could be a treatment option in advanced EMC [9]. A recent phase 2 study showed that pazopanib had clinically meaningful antitumor activity in patients with progressive and advanced EMC, and therefore could be considered a suitable option after failure to respond to first-line anthracycline-based chemotherapy in these patients [10]. Given the desperate need of new therapies for sarcomas, accurate preclinical models are essential to gain better molecular insights into pathogenesis of sarcomas and to develop new therapeutic approaches. Therefore, representative preclinical sarcoma models are required especially in the light of the exponentially growing number of compounds and possible compound combinations. To our knowledge there is only one cell line established in 1992 from an EMC, named *H-EMC-SS* [11]. The molecular profile is not available for this model and functional testing is lacking. Here, we present two novel ex vivo EMC models (*USZ20-EMC1 and USZ22-EMC2*) that were molecularly characterized and functionally tested for drug sensitivities and drug synergies.

## Materials and methods

### Patient information

*USZ20-EMC1:* A 54-year-old female patient initially presented in 2009 with a mass in her knee para-patellar medial right and was diagnosed with an extraskeletal myxoid chondrosarcoma (EMC). After several resections from loco-regional recurrences, the patient underwent an amputation of her lower leg in late 2020 and the *USZ20-EMC1* cell model was established from fresh material. The patient is currently suffering from metastatic disease to the lung. Lung metastases were continuously treated with surgical resection, radiation therapy and cryoablation. The *USZ22-EMC2* cell model was established from a diagnostic biopsy specimen of a 68 year old male patient that presented with a 13 cm mass in the left thigh. The present study was conducted following regional/cantonal and institutional guidelines and in compliance with the Helsinki Declaration and after approval by our cantonal ethical review board Zurich (BASEC-2021–00,417).

### Ex vivo cell model establishment

Fresh tumor tissue was obtained from the amputation specimen for *USZ20-EMC1 *ex vivo cell model and from a fresh diagnostic biopsy for *USZ22-EMC2 *ex vivo cell model development. The tumor tissues were mechanically dissected into small pieces and treated with liberase™ (TM Research Grade From Merck) at a concentration of 1 mg/mL for 4 h. The cells were plated in 6 well ultra low attachment plates (ULA; Corning) and maintained in a 1:1 mix of advanced Dulbecco’s modified Eagle’s medium (Thermo Fisher Scientific, Waltham, MA, USA) supplemented with 10% heat-inactivated horse serum (Gibco), 1 × Glutamax (Gibco), 100 μg/ml primocin (Invivogen) mixed with CHK Media containing¸ B27 supplement (Gibco), 1.25 mM N-acetylcysteine (Sigma-Aldrich), 50 ng/ml human recombinant EGF (ThermoFisher Scientific), 20 ng/ml human recombinant FGF-10 (ThermoFisher Scientific), 1 ng/ml recombinant human FGF-basic (ThermoFisher Scientific), 500 nM A-83-01 (Tocris Bioscience, Birston, UK), 10 μM SB202190 (Selleck Chemical Inc), 10 mM Nicotinamide (Sigma-Aldrich), 1 μM PGE2 (Sigma-Aldrich), 25 nM Hydrocortisone (HC, Sigma-Aldrich), 0.5 μg/ml epinephrine (Sigma-Aldrich) and R-Spondin (conditioned media, self-produced). The sarco-spheres were passaged every 2–3 weeks with a gentle digestion using TryLE (Gibco) in a water bath at 37 °C and transferred to another tissue culture plate. The cell culture was incubated at 37 °C in a humidified atmosphere with 5% CO_2_. Both cell models were able to attach to collagen I-coated plates and grow in 2D as a monolayer culture.

### Cell proliferation assay

To determine the growth rate of both established cell models, 5,000 cells/well were seeded in 96-well plates (Corning, USA) in 12 replicates and incubated at 37 °C and 5% CO_2_. At day 0, 4, 8 and 12 after seeding, cell proliferation was assessed by CellTiter-Glo® viability assay (Promega, Madison, WI) according to the manufacturer’s protocol. Luminescence signal was read with the Infinite 200 Pro (Tecan). Growth curves were plotted as fold of change of relative luciferase units (RLU) on each time point to calculate the doubling time for each cell model using GraphPad PRISM.

### Mycoplasma contamination detection

100 µl supernatant was taken from cell cultures reached 90% confluence, followed by 5 min incubation at 95 °C and 5 s centrifugation at 13,000 rpm. 2 µl was used for PCR reactions using PCR Mycoplasma Test Kit II(AppliChem GmbH, Germany) according to manufacturer’s protocol. DNA unique to mycoplasma rRNA operon was amplified. Internal control and positive control DNA and primers were included in the PCR reaction. PCR products were separated with a 1.5% standard agarose gel and imaged with Chemidoc XRS + (Bio-Rad). ImageLab software (Bio-Rad) was used for imaging analysis.

### Molecular characterization of the ex vivo cell models

*Next generation sequencing*: FoundationOne®HEME assay is a next generation sequencing (NGS) assay that uses a hybrid capture methodology and detects base substitutions, insertions, deletions, and copy number (CN) alterations in up to 406 genes and gene rearrangements in up to 265 genes, tumor mutation burden and microsatellite instability using the previously described methods [12]. DNA and RNA was extracted using the Maxwell® Tissue DNA Purification Kit (Promega AS1030). Library construction was done using NEBNext kits (NEB E6040S) and the sequencing was performed on a HiSeq2500 according to clinical laboratory standards with 150-base pair paired-end reads (Department of Pathology and Molecular Pathology, University Hospital Zurich, Switzerland).

*Methylation and copy number analysis:* 500 ng genomic DNA (from the same extraction as for NGS) were subjected to bisulfite conversion using a validated in house protocol from our clinical laboratory (Department of Pathology and Molecular Pathology, University Hospital Zurich, Switzerland). The Infinium Human Methylation EPIC BeadChip (850 K) array was used to obtain genome wide DNA methylation profiles according to the manufacturer’s instructions (Illumina, USA). The quality of each sample was checked using the on-chip quality metrics and the R package *minfi* version 1.40 [13]. IDAT files were uploaded to the DKFZ Sarcoma Classifier (version 12) *(*www.molecularsarcomapathology.org*)* to validate the diagnosis and the models. Classifier results consisted of a suggested methylation class with an accompanying calibrated score. The calibrated score is a probability of the confidence for the given methylation class assignment. As defined by Koelsche et al. the classifier was only deemed to have made a successful prediction if the sample obtained a calibrated score of 0.9 or higher [14]. Both samples showed a score above 0.9 and matched to the entity classified as extracellular myxoid chondrosarcoma.

*Fluorescence *in situ* hybridization (FISH): NR4R3* FISH for both cell models was conducted on 2 μm thick sections using dual color break apart (bap) FISH probes for *NR4R3 (*Abnova™ Thermo Fisher*)* according to the manufacturer's protocol.

### Authentication and quality control of both established cell models

Both established cell models and the corresponding native tumor material were additionally authenticated by examining highly polymorphic short tandem repeats (STRs) using the PowerPlex®16 HS System (Promega) according to the manufacturer’s instructions. Fragment analysis was done on an ABI3730xl (Life Technologies) and the STR patterns were analyzed by the GeneMapper software (Thermo Fisher Scientific) and matched to the data in the public cell banks using a function of Cellosaurus with a standard match threshold of 80%.

### Drug screening

A medium throughput drug screen using 40 drugs was conducted with *USZ20-EMC1* at passage 5. Eight-hundred cells per well were seeded into 384-well ULA plates (Corning) containing 50 μl media. A drug library including 23 targeted agents and 17 chemotherpies was acoustically administered (Labcyte Echo) as single agents using contactless, nanovolume liquid transfers to create a 3-log, 6-dose drug curves; drug concentrations ranged from 33 pmol/l to 200 μmol/l. Sarco-spheres were challenged with drugs for 6 days, and subsequently relative viability was determined by whole-well ATP quantification using Cell-Titer-Glo 2.0 (Promega) and normalized to vehicle-only controls (maximal DMSO concentration used was 0.2%). The AUC for drug response was used as this metric combines information about the efficacy (how much cell viability is decreased by each drug) and potency (the amount of drug needed to reduce viability; IC_50_) of each drug [15]. Drug sensitivities for carfilzomib, doxorubicin and venetoclax were validated in a 96 well format in triplicates for both cell models *USZ20-EMC1* and *USZ22-EMC2*. 1000 cells per well were seeded, and 24 h after plating, drugs were tested in a 6-point dilution dose response log scale to determine the IC_50_ values. The highest dose used was 10 µM and lowest dose was 4.7 pM. Furthermore, combination treatments of these drugs were investigated in a 5-point dilution dose response in log scale, ranging from 10 µM to 1 nM for doxorubicin, and 1 µM to 100 pM for carfizomib and venetoclax. CellTiter Glo 2.0 (Promega) was added to the cells for the endpoint read out at day 6. Plates were read using the Infinite 200 Pro from Tecan at 570 nm. Analysis was performed running a non-linear regression (curve fit) method in GraphPad Prism 9.1.1 (GraphPad Software, San Diego, CA, USA). Drug synergies were analyzed using the *synergyfinder* package [16]. *Synergyfinder* provides efficient implementations for all the popular synergy scoring models, including HSA, Loewe, Bliss and ZIP.

## Results

### Establishment of two novel ex vivo exraskeletal myxoid chondrosarcoma (EMC) sarco-sphere cell models (USZ20-EMC1 and USZ22-EMC2)

**Fig. 1:**
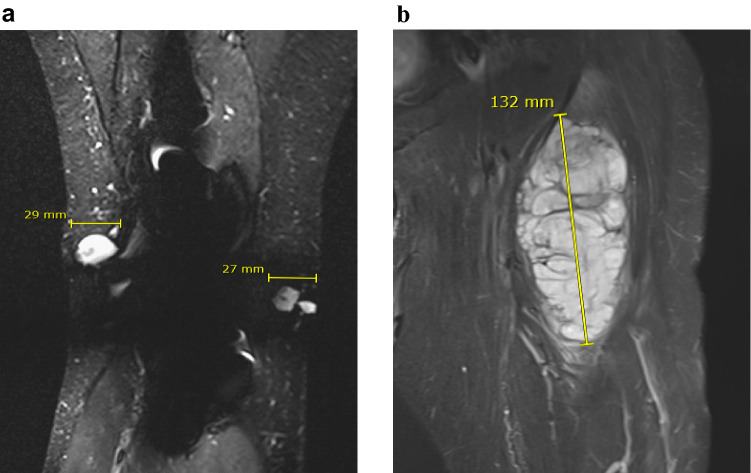
Magnetic resonance imaging shows recurrent tumor nodules around the knee prosthesis in (**A)**, this tumor has been the source of *USZ20-EMC1.* A deep-seated multilocular tumor in the left thigh (**B)** high intensity on T2-weighted images has been the source for *USZ22-EMC2*

**Fig. 2:**
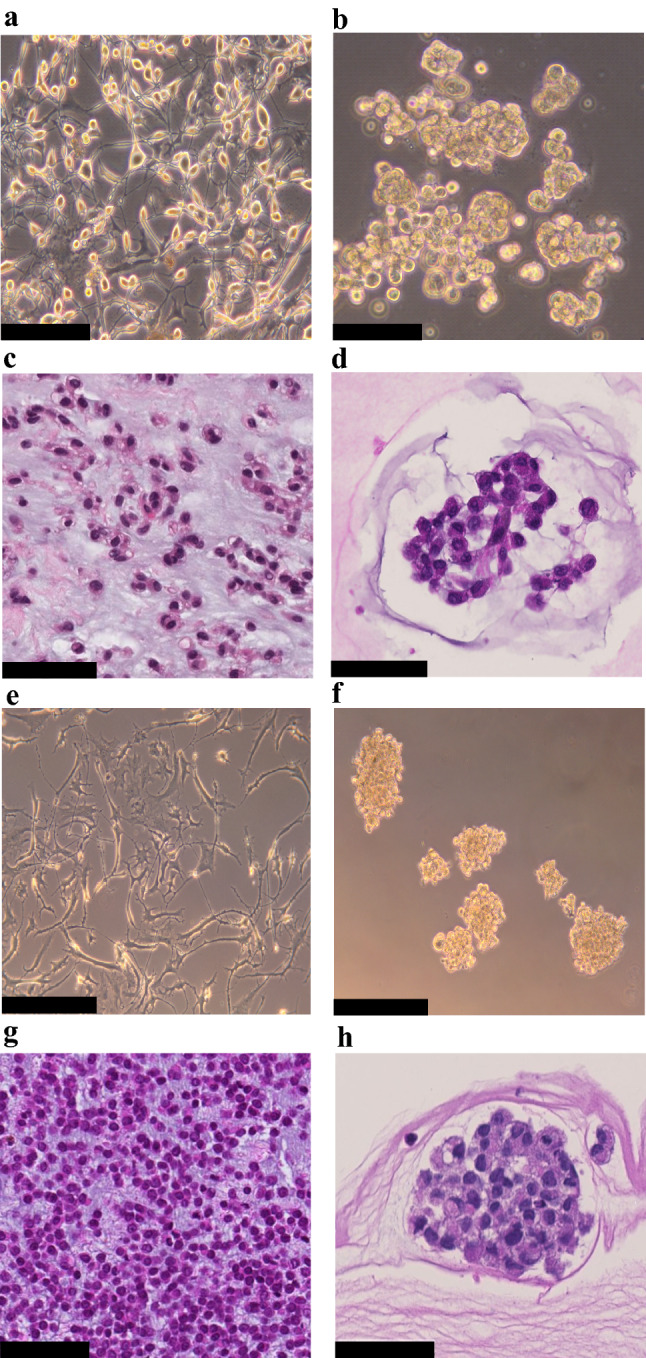
Bright field images show cells attached as a monolayer culture in 2D for *USZ20-EMC1* (**A)** and for *USZ22-EMC2* **(E**) and grown as sarco-spheres in 3D for *USZ20-EMC1* (**B)** and for *USZ22-EMC2* **(F**). Hematoxylin and Eosin stains show in the native tumor tissue (**C, G**) cells that are embedded in an abundant pale-blue myxoid or chondromyxoide matrix. The cells themselves have a deeply eosinophilic cytoplasm, as well as uniform round to oval nuclei. Corresponding cell morphology is seen in the histology from the sarcosphere models native tumor and correscponsing organoids (**D, H**). **C + D** are representing tissue and sarco sphere from *USZ20-ECM1* and **G + H** are representing tissue and sarco sphere from *USZ22-ECM2*. Scale bars indicate 50 µM in C, D, G and H, 100 µM in A, B, E and F

**Fig. 3:**
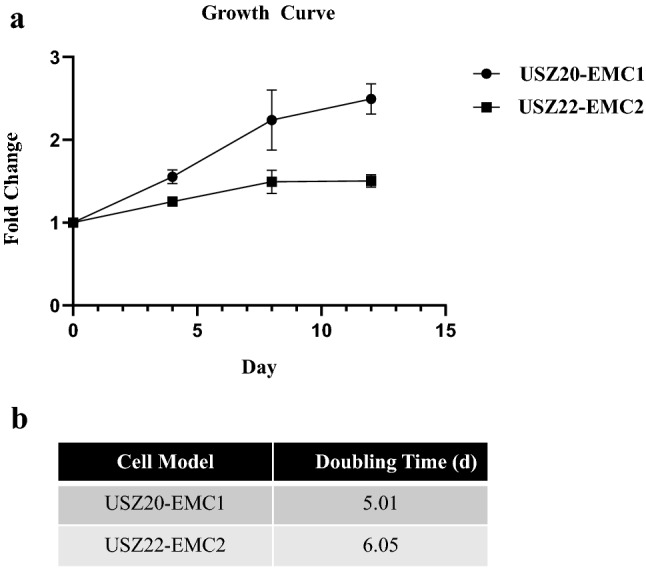
Cell proliferation was assed at day 0, 4, 8 and 12 after seeding using CellTiter-Glo® viability assay (Promega, Madison, WI) according to the manufacturer’s protocol. Growth curves were constructed by plotting fold of change of Relative Luciferase Units (RLU) on each time point to calculate the doubling time for each cell line using GraphPad PRISM (**A**). Doubling time for *USZ20-EMC1* was assessed as 5.09 days and 6,05 days for *USZ22-EMC2* (**B**)

We established two novel ex vivo sarco-sphere cell models from two patients diagnosed with an EMC. *USZ20-EMC1* was established from an amputation specimen from a 54 year old patient with a long history of recurrent and metastatic EMC and *USZ22-EMC2* was established from a fresh biopsy taken from a left thigh mass of a 68 year old patient at the time of diagnosis (Fig. 1a, b). *USZ20-EMC1* and *USZ22-EMC2* cells were cultured in 3D as sarco-spheres for more than 24 passages over 12 months and more than 8 passages over 6 months, respectively (Fig. 2b, f). Cells were biobanked in our living biobank in the Department of Pathology and Molecular Pathology, University Hospital Zurich, Switzerland. Both models were also able to attach and grow on collagen coated plates as monolayer cultures (2D) (Fig. 2a, e). Phenotypic analysis showed that the morphology of the sarco-sphere models recapitulated the native tumor tissue (Fig. 2c, d, g, h). Inference of their cell growth indicated a doubling time of 5.09 days for *USZ20-EMC1* and 6.05 days for *USZ22-EMC2* (Fig. 3a, b).

### Molecular characterization of USZ20-EMC1 and USZ22-EMC2

**Fig. 4:**
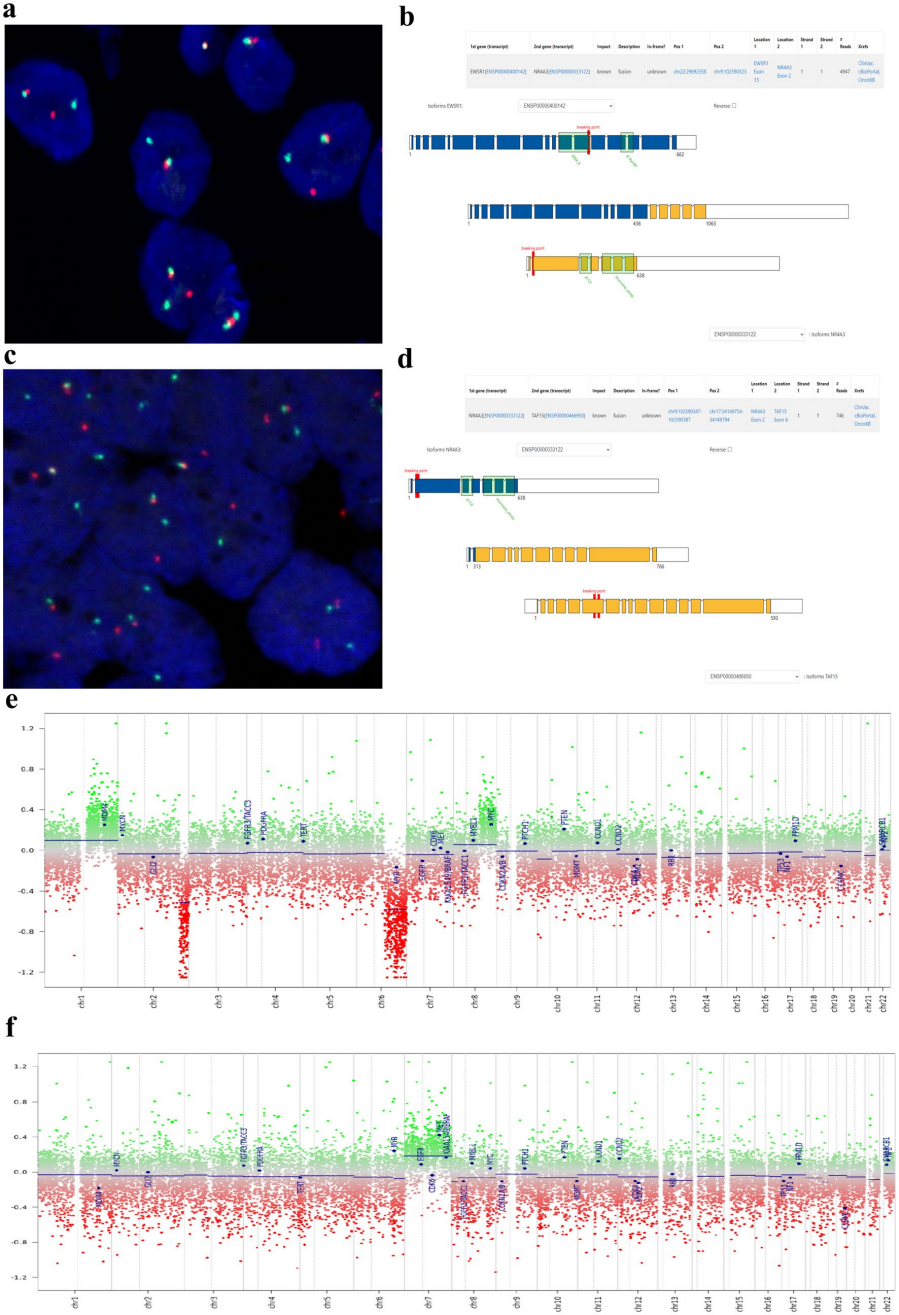
Both cell models show a break apart signal in the fluorescence in situ hybridization (FISH) indicating the rearrangement for the *NR4R3* gene, for *USZ20-EMC1* (**A) **and *USZ22-EMC2* **(C**). The rearrangement and fusion partner was confirmed by NGS using the FoundationOne®HEME assay. For *USZ20-EMC1; EWSR1* was confirmed as fusion partner having exon 13 for *EWSR1* on chr22 and exon 2 from *NR4A3* on chr9 involved (**B**). For *USZ22-EMC2; TAF15* was confirmed as fusion partner having exon 6 for *TAF15* on chr17 and exon 2 from *NR4A3* on chr9 involved (**D**). Copy number profiles (CNV) show specific gains and losses on chromosomes 1, 2, 6 and 8 for both cell models, *USZ20-EMC1* shown in **E** and *USZ22-EMC2* in **F**

FISH analysis for *NR4A3* confirmed a rearrangement in both established ex vivo cell models at a passage 8 (Fig. 4a, c). We complemented the genomic profiling using the FoundationOne®HEME assay for both ex vivo cell models at passage 8. For *USZ20-EMC1*, an *EWSR1-NR4A3* rearrangement and, for *USZ22-EMC2*, a *TAF15-NR4A3* rearrangement in the native tumor tissue and the corresponding cell model was confirmed on the RNA level (Fig. 4b, d). Tumor and sarco-spheres for both samples showed low tumor mutational burden (TMB) (< 5 mut/MB) and a stable microsatellite status (MS-stable). For *USZ20-EMC1* on the DNA level multiple genomic short-variant mutations were detected that have been classified as variants of unknown significance (VUS). *USZ22-EMC2* harbors two additional likely pathogenic mutations in *MLL3* and *KDM5C* beside a *FANCA* alteration that has been classified as a VUS. Details about these alterations are listed in the Supplementary Table 1. DNA methylation profiling was performed from cells at passage 8 and confirmed the methylation class for EMC with a score of 0.99 for both cell models using the DKFZ Sarcoma Classifier platform version 12 (www.molecularsarcomapathology.org). The copy number profiles exhibited gains mainly in chromosome 1, where the *MDM4* locus is located, and in chromosome 8, where *MYC* is located for *USZ20-EMC1. USZ22-EMC2* showed a chromosome 8 amplification with for example EGFR and MET localized and losses were identified in chromosome 2 and 6, where for example MYB is located (Fig. 4e, f). Beside these few copy number alterations, there was a rather flat CNV plot for both models despite of the fusion partner as the driver alteration. Based on our genomic and methylation analysis we confirmed that the cell models are suitable ex vivo models that represent the native tumor tissue. We further authenticated both cell models by analyzing highly polymorphic short tandem repeats (STR) of 16 microsatelllites and confirmed identical STR allel patterns between the native tumor and corresponding cell model. Both STR patterns did not mach those of any other cell line abvaialbel with within public cell banks examined using the cell line database, Cellosaurus (Supplementary Table 2). To our knowledge, *USZ20-EMC1* and *USZ22-EMC2* are so far the only moleculary well-characterized ex vivo cell models for EMC.

### Functional characterization of USZ20-EMC1 and USZ22-EMC2 uncovers novel drug sensitivities and synergistic drug combinations

**Fig. 5:**
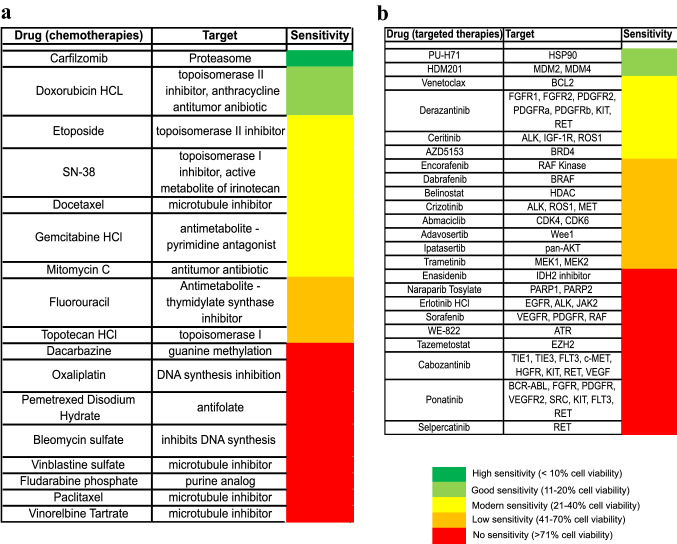
Heat map indicating drug response divided in high (< 10% cell viability), good (11–20% cell viability), moderate (21–40% cell viability), low (41–70% cell viability) or no (> 71% viability) sensitivity is shown for 17 chemotherpies (**A**) and 23 targeted agents (**B**) for *USZ20-EMC1.* Drug efficacy (how much cell viability is decreased by each drug) and potency (the amount of drug needed to reduce viability; IC_50_) were analyzed using the AUC

**Fig. 6:**
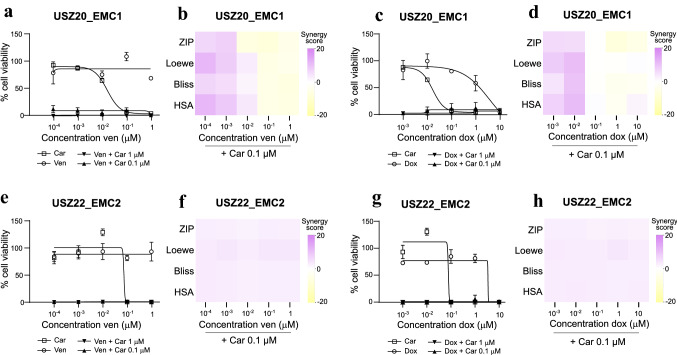
Sensitivity of EMC cell models to carfilzomib in combination with venetoclax or doxorubicin. Ex vivo treatment of *USZ20-EMC1* (**A**) and *USZ22-EMC2* (**E**) sarco-spheres for 6 days with five doses of the proteasome inhibitor carfilzomib and the BCL-2 inhibitor venetoclax as single agents or in combination (venetoclax plus 1 µM or 0.1 µM carfilzomib). Heatmap of the synergy scores ZIP, Loewe, Bliss and HSA showing synergy of 0.1 µM carfilzomib with low doses of venetoclax (**B**) or additive effects (**F**) in the combinatorial modality. Ex vivo treatment of *USZ20-EMC1* (**c**) and *USZ22-EMC2* (**G**) sarco-spheres for 6 days with five doses of the proteasome inhibitor carfilzomib and the anthracycline drug doxorubicin as single agents or in combination (doxorubicin plus 1 µM or 0.1 µM carfilzomib). Heatmap of the synergy scores ZIP, Loewe, Bliss and HSA showing synergy of 0.1 µM carfilzomib with low doses of doxorubicin (**D**) or additive effects (**H**) in the combinatorial modality. Data are mean ± s.d

To evaluate individual drug responses, we subjected sarco-spheres at p6 from *USZ20-EMC1* to a medium-throughput drug–dose response screening. Sarco-spheres were challenged with a 40 pan-cancer drug panel consisting of both chemotherapies (*N* = 17) and targeted agents (N = 23) (Fig. 5a, b). Drug sensitivities were classified as (i) none, (ii) low to moderate and (iii) good to high. From the 17 tested chemotherapeutic drugs, carfilzomib, a proteosome inhibitor was the only compound that showed high sensitivity, followed by doxorubicin with good sensitivity. All the other chemotherapeutics did only show moderate, low or no sensitivity (Fig. 5a). Similar results we found for the 23 tested targeted agents. PU-H71 (HSP90) and HDM201 (MDM2/MDM4) performed best from the compounds tested and showed good sensitivity while the cells had a moderate sensitivity to venetoclax. In general there was none to only moderate sensitivity for the most screened targeted therapeutics tested in *USZ20-EMC1* (Fig. 5b). Screening results of higher relevance were validated and reproduced for calfilzomib, doxorubicin, and venetoclax with dose response curves in both cell models within passage 8 and 10. Both models independent of the *NR4A3* fusion partner showed high sensitivity to carfilzomib and good to moderate sensitivity to doxorubicin, while there was no response to venetoclax as a monotherapy in the validation (Fig. 6a, c, e, g). Drug synergies were further investigated in a combinatorial modality by pairing carfilzomib with venetoclax and carfilzomib with doxorubicin. We found dose-dependent additive and synergistic effects in both cell models for carfilzomib in combination with venetoclax and doxorubicin (Fig. 6a–h). Drug synergy was found for *USZ20-EMC1* cell models using both combinations according to the ZIP, Loewe, Bliss and HSA score using Synergy finder and an additive effect in the *USZ22-EMC2* model (Fig. 6b, d, f, h). Similar drug responses were seen in both models, independent of the fusion partner from *NR4A3.*

## Discussion

Patient-derived cancer ex vivo cell models are valuable tools in the era of functional precision medicine where drugs can directly be tested to guide patient care. Furthermore, physiological ex vivo models are important tools for basic research and preclinical translational studies to study novel drug targets. As such models slowly get integrated into functional precision oncology and clinical decision making, it is of utmost importance that these ex vivo models represent the native tumor on a phenotypic and genotypic level. EMC represents an exceptionally rare sarcoma subtype with uncertain differentiation. As other sarcoma subtypes, EMC often displays chemotherapy resistance and new therapeutic options are urgently needed. Drug screening studies have been conducted using a number of cell lines and more recently patient derived ex vivo 3D cell models to identify novel candidate drugs and novel drug combinations for common cancer types. However, due to the lack of EMC ex vivo cell models, up to date no drug screening has been conducted for this sarcoma subtype. There is only one EMC cell line (H-EMC-SS) reported in the literature, established in 1992 [11]. Proper molecular profiling was not conducted with this model. Due to the lack of well-chararcterized EMC models, studies of drug sensitivity to anti-cancer agents are lacking. To the best of our knowledge, we here present the first molecularly characterized and functionally tested ex vivo EMC models (*USZ20-EMC1* and *USZ22-EMC2*). The *USZ20-EMC1* model was established from recurrent tumor tissue from a 54 year old patient while *USZ22-EMC2* was established from a diagnostic biopsy taken from a 68 year old patient. Genomic and methylation profiling confirmed the *EWSR1-NR4A3* and the *TAF15-NR4A3* rearrangement and the diagnoses of both models. Drug screening identified limited sensitivities to most chemotherapeutic and targeted agents as kind of expected based on current clinical knowledge. Remarkably, carfilzomib (a proteasome inhibitor) showed the highest sensitivity among the tested drugs and was considered a promising candidate. Proteasome inhibitors, such as bortezomib and carfilzomib, have shown efficacy in anti-cancer therapy mainly in hematological diseases but not in solid cancers. The combination of carfilzomib and doxorubicin has shown promising results in clinical trials for relapsed and refractory multiple myeloma [17, 18]. Recently, sentitivity to proteasome inhibition was found in liposarcoma. The authors described synergistic effects between carfilzomib and selinexor (an XPO1-mediated nuclear export inhibitor) [19]. Furthermore, carfilzomib sensitivity was explored in pediatric tumors such as neuroblastoma, ewing sarcoma, osteosarcoma, rhabdomyosarcoma and atypical teratoid rhabdoid tumor (ATRT). Synergistic effects were reported in these models with carfilzomib in combination with chemotherapeutic drugs of different classes [20]. For the tested chemotherapeutic drugs, doxorubicin monotherapy displayed good sensitivity in our two models. Doxorubicin is commonly used in STS in need for systemic treatment. Based on published data in other tumor entities, we investigated whether carfilzomib and doxorubicin could show a synergistic effects in ex vivo EMC models. We confirmed a dose dependant synergistic effect in one and an additive effect in the other ex vivo cell model using Synergy finder. Regarding targeted agents, we observed in the medium throughput screen a good sensitivity for HSP90 inhibitor PU-H71, which is a drug that also has been explored in Ewing sarcoma ex vivo [21]. Venetoclax showed a moderate response in the *USZ20-EMC1* high medium screen but not in the validation as monotherapy in both models. As venetoclax and doxorubicin both showed promising results in combination for relapsed and refractory multiple myeloma we investigated this combinations for both EMC models [22]. Interestingly, we found these compounds in combination to exhibit a dose-dependent additive and synergistic effects that were especially pronounced at a lower dose of doxorubicin. Based on our data, carfilzomib might be a promising candidate drug for EMC mono- or combination therapy, mimicking responses observed for hematological disease. In conclusion, we generated two novel ex vivo EMC cell models with thorough molecular and phenotypic characterization. *USZ20-EMC1* and *USZ22-EMC2* cells exhibited constant proliferation as sarco-sphere models. We identified novel drug sensitivities for monotherapy and novel synergistic drug combinations independent of the fusion partner. Further studies are required to investigate the underlying pharmacological mechanisms an the in vivo efficacy of the here identified combination treatments. The here described cell models can further be used to study molecular consequences of the *NR4A3* gene fusions, especially in regards to the fusion partners *EWSR1* (Ewing Sarcoma RNA Binding Protein 1) and/or *TAF15* (TATA-Box Binding Protein Associated Factor 15).

## Supplementary Information

Below is the link to the electronic supplementary material.

Supplementary file1 (PDF 89 KB)

Supplementary file2 (PDF 108 KB)

## Data Availability

The datasets used and/or analyzed during the current study are available from the corresponding author on reasonable request. Both cell models *USZ20-EMC1* and *USZ22-EMC2* can be made available from the Laboratory for Systems Pathology and Functional Tumor Pathology, Department for Pathology and Molecular Parhology, University of Zurich, Zurich.
