# Supplementary material for: Establishment, characterization and functional testing of two novel ex vivo extraskeletal myxoid chondrosarcoma (EMC) cell models
Source: Hum Cell. 2022 Nov 1;36(1):446–55. doi: 10.1007/s13577-022-00818-x (PMC9813045; doi:10.1007/s13577-022-00818-x)
Supplement: Supplementary file 1 — Supplementary file1 (PDF 89 KB) [file 13577_2022_818_MOESM1_ESM.pdf]

Supplementary Table 1: Additional molecular alteration on a DNA level			
**ID**	**VARIANT-TYPE**	**GENE**	**SOMATIC STATUS - FUNCTIONAL IMPACT**
USZ20-EMC1	Tumor content: 50%		
	Tumor Mutational Burden		
	Microsatellite Status		
	short-variant	KIT	unknown
	short-variant	AKT2	unknown
	short-variant	TBL1XR1	unknown
	short-variant	PIK3R2	unknown
	short-variant	NCOR2	unknown
	short-variant	MYST3	unknown
	short-variant	INPP5D	unknown
	short-variant	HSP90AA1	unknown
	short-variant	GNAS	unknown
USZ22-EMC2	Tumor content: 90%		
	Tumor Mutational Burden		
	Microsatellite Status		
	short-variant	MLL3	likely
	short-variant	KDM5C	likely
	short-variant	FANCA	unknown

SV-PROTEIN-CHANGE	SV-CDS-CHANGE	SV-GENOME-POSITION	SV-COVERAGE
L455V	1363C>G	chr4:55592039	1083
G116A	347G>C	chr19:40748535	459
N160H	478A>C	chr3:176768348	946
V54M	160G>A	chr19:18266849	447
E2521K	7561G>A	chr12:124809953	369
P1661_P1662insQQPQP	4982_4983insACAGC	chr8:41790755	577
T532S	1595C>G	chr2:234077993	606
L79F	235C>T	chr14:102568343	852
Q267*	799C>T	chr20:57484819	916
E1689fs*28	5065delG	chr7:151882659	1351
splice site 1584-2A>T	1584-2A>T	chrX:53239760	377
A40V	119C>T	chr16:89882355	726

**SV-PERCENT-READS**	**BIOMARKER-VALUE**
	1.36 Muts/Mb
	MS-Stable
50.05	
46.62	
47.78	
48.32	
53.39	
38.65	
46.37	
48.94	
9.28	
	1.7 Muts/Mb
	MS-Stable
19.54	
13.00	
49.86	
