# Supplementary material for: Establishment, characterization and functional testing of two novel ex vivo extraskeletal myxoid chondrosarcoma (EMC) cell models
Source: Hum Cell. 2022 Nov 1;36(1):446–55. doi: 10.1007/s13577-022-00818-x (PMC9813045; doi:10.1007/s13577-022-00818-x)
Supplement: Supplementary file 2 — Supplementary file2 (PDF 108 KB) [file 13577_2022_818_MOESM2_ESM.pdf]

**Supplementary Table 2: Short tandem repeat analysis**

| Locus | Chromosome Location | *USZ20-EMC1* Tumor tissue Typed allel | *USZ20-EMC1* Cell model Typed allel | Databases Alleles |
|---|---|---|---|---|
| Amelogenin | X | X | X | N/A |
| D3S1358 | Chr03 | 15/17 | 15/17 | N/A |
| TH01 | Chr11 | 7/9.3 | 7/9.3 | N/A |
| D21S11 | Chr21 | 30 | 30 | N/A |
| D18S51 | Chr18 | 15 | 15 | N/A |
| Penta_E | Chr15 | 5/10 | 5/10 | N/A |
| D5S818 | Chr05 | 9/13 | 9/13 | N/A |
| D13S317 | Chr13 | 9/13 | 9/13 | N/A |
| D7S820 | Chr07 | 8/10 | 8/10 | N/A |
| D16S539 | Chr16 | 12 | 12 | N/A |
| CSF1PO | Chr05 | 10/11 | 10/11 | N/A |
| Penta_D | Chr21 | 13/15 | 13/15 | N/A |
| vWA | Chr12 | 14/16 | 14/16 | N/A |
| D8S1179 | Chr08 | 12/13 | 12/13 | N/A |
| TPOX | Chr2 | 8 | 8 | N/A |
| FGA | Chr04 | 22 | 22 | N/A |
| **Locus** | **Chromosome Location** | ***USZ22-EMC2* Tumor tissue Typed allel** | ***USZ22-EMC2* Cell model Typed allel** | **Databases Alleles** |
| Amelogenin | X/Y | X/Y | X/Y | N/A |
| D3S1358 | Chr03 | 14/15 | 14/15 | N/A |
| TH01 | Chr11 | 7/9.3 | 7/9.3 | N/A |
| D21S11 | Chr21 | 29 | 29 | N/A |
| D18S51 | Chr18 | 12/13 | 12/13 | N/A |
| Penta_E | Chr15 | 12/13 | 12/13 | N/A |
| D5S818 | Chr05 | 10/12 | 10/12 | N/A |
| D13S317 | Chr13 | 11/14 | 11/14 | N/A |
| D7S820 | Chr07 | 10 | 10 | N/A |
| D16S539 | Chr16 | 11/12 | 11/12 | N/A |
| CSF1PO | Chr05 | 11/12 | 11/12 | N/A |
| Penta_D | Chr21 | 12/13 | 12/13 | N/A |
| vWA | Chr12 | 15/16 | 15/16 | N/A |
| D8S1179 | Chr08 | 10/13 | 10/13 | N/A |
| TPOX | Chr2 | 8/9 | 8/9 | N/A |
| FGA | Chr04 | 22 | 22 | N/A |
